# Polyamine metabolism and anti-tumor immunity

**DOI:** 10.3389/fimmu.2025.1529337

**Published:** 2025-02-18

**Authors:** Jing-Yi Wu, Yan Zeng, Yu-Yang You, Qi-Yue Chen

**Affiliations:** ^1^ Fujian Medical University, Fuzhou, Fujian, China; ^2^ Department of Gastric Surgery, Fujian Medical University Union Hospital, Fuzhou, China

**Keywords:** polyamine, tumorigenesis, polyamine combination therapeutic strategies, metabolism, tumor microenvironment

## Abstract

Growing attention has been directed toward the critical role of polyamines in the tumor microenvironment and immune regulation. Polyamines, primarily comprising putrescine, spermidine, and spermine, are tightly regulated through coordinated biosynthesis, catabolism, and transport, with distinct metabolic patterns between normal and cancerous tissues. Emerging evidence highlights the pivotal role of polyamine metabolism in tumor initiation, progression, and metastasis. This review aims to elucidate the differences in polyamine biosynthesis, transport, and catabolism between normal and cancerous tissues, as well as the associated alterations in tumor epigenetic modifications and resistance to immune checkpoint blockade driven by polyamine metabolism. Polyamine metabolism influences both tumor cells and the tumor microenvironment by modulating immune cell phenotypes—shifting them towards either tumor suppression or immune evasion within the tumor immune microenvironment. Additionally, polyamine metabolism impacts immunotherapy through its regulation of key enzymes. This review also explores potential therapeutic targets and summarizes the roles of polyamine inhibitors in combination with immunotherapy for cancer treatment, offering a novel perspective on therapeutic strategies.

## 1 Background

Tumor cells exhibit defining traits such as limitless proliferative capacity, immune evasion, and metastasis. Alterations in metabolic patterns are pivotal to the development, growth, and progression of tumors (1). In the tumor microenvironment, cancer cells experience metabolic reprogramming encompassing polyamine metabolism. Polyamine metabolism is the metabolic process involving the intracellular polyamines from their synthesis to their catabolism. Polyamines are small organic molecules containing two or more amino groups that are crucial for cell growth, division, gene regulation, DNA stability and protein synthesis. They are vital for fundamental biological processes that contribute to the control of immune cells, including immune cell regulation, signaling pathway modulation, cell proliferation and differentiation, and development (2). Dysregulation of polyamine metabolism is prevalent in neoplastic tissues, exhibiting elevated polyamine concentrations compared to normal tissues. Furthermore, the increase of polyamines inhibits immune cell activity and facilitates tumor progression. Reducing polyamine levels, either via pharmacological agents or gene deletion, can result in a deceleration of tumor proliferation. This indicates that polyamine metabolism may serve as a therapeutic target for cancer treatment. A growing number of people are interested in the function of polyamines in malignant tumors and polyamine-targeted pharmaceuticals (3). This study investigates the mechanisms by which polyamine metabolism modulates tumor immunity and explores therapeutic strategies targeting polyamine metabolism.

## 2 Polyamine metabolism in normal and cancer cells

In mammals, polyamine levels are controlled by a coordinated balance of biosynthesis, transport, and catabolism. In malignant tumors, the accumulation of polyamines typically arises from the dysregulation of these three components.

### 2.1 Polyamine metabolism in normal tissues

#### 2.1.1 Biosynthesis

**Figure 1:**
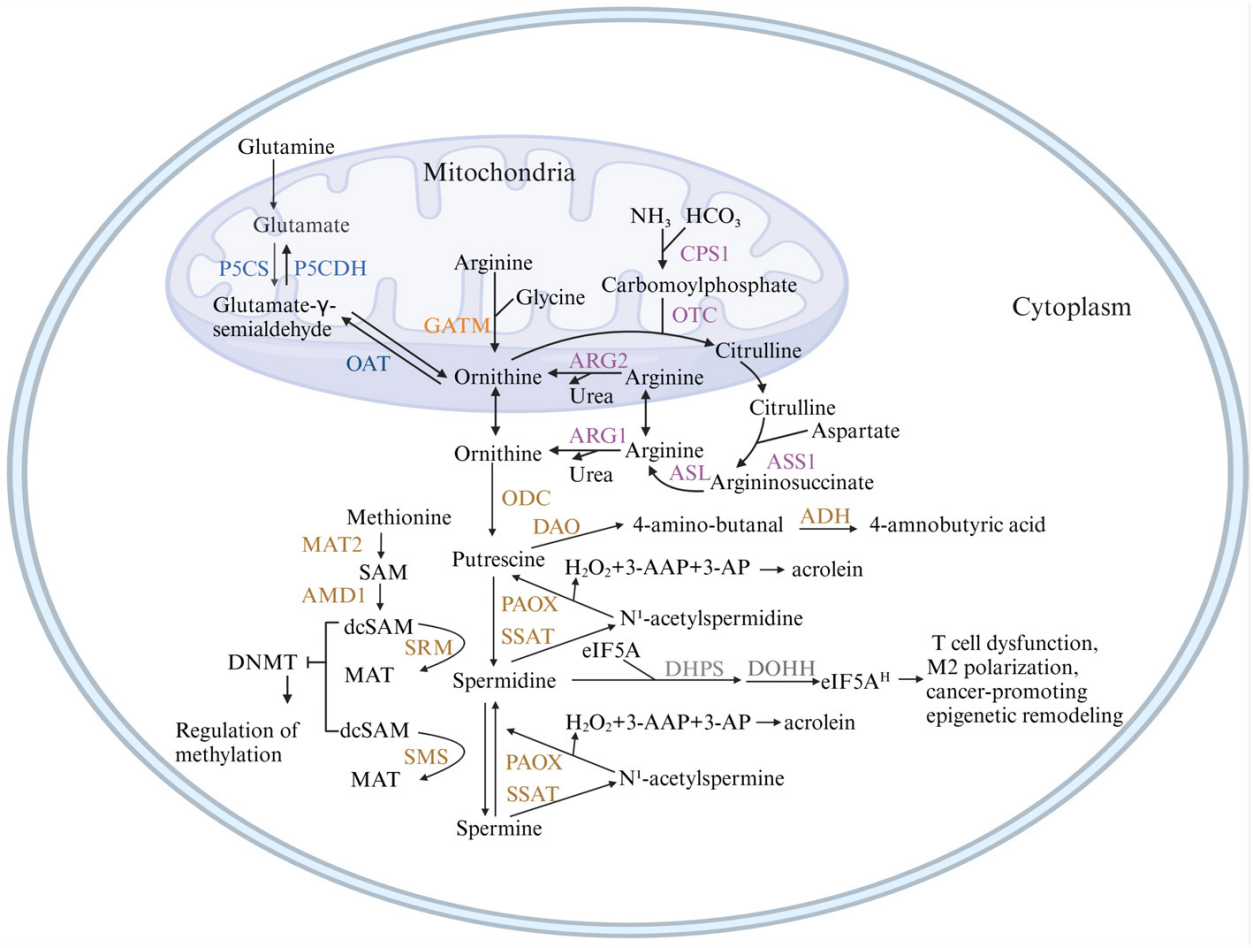
Metabolic pathways of polyamines. This figure illustrates the pathways of polyamine synthesis and catabolism. Ornithine, the precursor of polyamines, can originate from three pathways: the *de novo* synthesis pathway (blue), which predisposes to cancer; the creatine synthesis pathway (orange); and the urea cycle pathway (purple), which are the primary sources of ornithine in normal tissues. Figure created using BioRender.com. ADH, aldehyde dehydrogenase; AMD1, adenosylmethionine decarboxylase 1; ARG, arginase; ASL, argininosuccinatelyase; ASS1, argininosuccinate synthase 1; CPS1, carbamoyl-phosphate synthase 1; DAO, diamine oxidase; DHPS, deoxyhypusine synthase; DOHH, deoxyhypusine hydroxylase; GATM, glycine amidinotransferase; MAT2, methionine adenosyltransferase; OAT, ornithine aminotransferase; ODC, ornithine decarboxylase 1; OTC, ornithine transcarbamoylase; P5CS, pyrroline-5-carboxylate synthase; P5CDH, pyrroline-5-carboxylate dehydrogenase; PAOX, polyamine oxidase; SMOX, spermine oxidase; SMS, spermine synthase; SRM, spermidine synthase; SSAT, spermidine/spermine N1-acetyltransferase 1.

As shown in **Figure 1**, arginine serves as the primary carbon donor for polyamine biosynthesis in organisms. Arginase (ARG) catalyzes the hydrolysis of arginine to form ornithine. In the presence of ornithine decarboxylase (ODC), ornithine is decarboxylated to produce putrescine. Subsequently, under the action of spermine synthase (SRM), putrescine is sequentially converted into spermidine and spermine. Both processes necessitate the provision of aminopropyl groups derived from decarboxylated S-adenosylmethionine (dcSAM), which is synthesized sequentially from methionine via methionine adenosyltransferase (MAT2) and adenosylmethionine decarboxylase 1 (AMD1) (4).

#### 2.1.2 Transport

**Figure 2:**
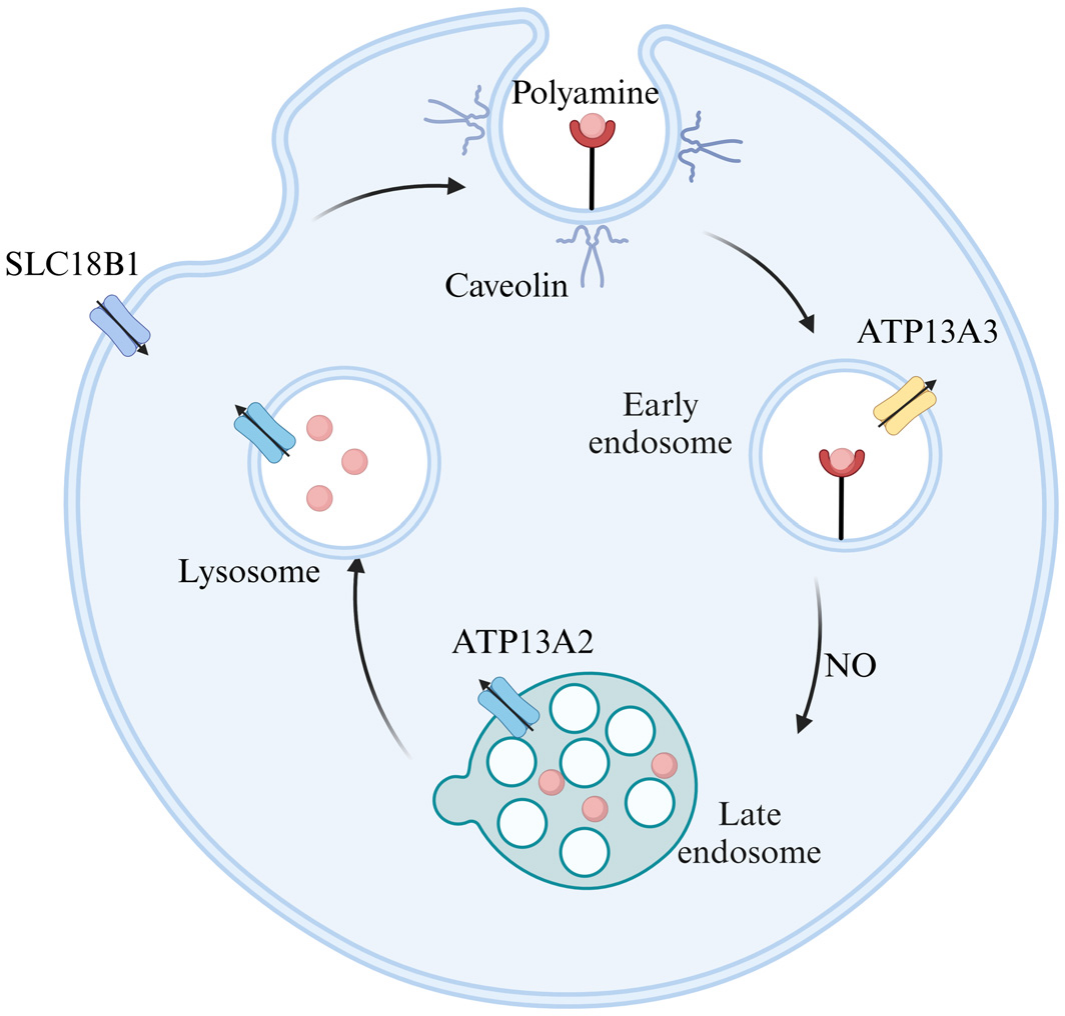
Models of polyamine transport. ATP13A2 exhibits the highest substrate specificity for spermine, followed by N1-acetylspermine and spermidine. ATP13A3 primarily transports putrescine, while SLC18B1 is responsible for spermine and spermidine, and SLC3A2 mainly carries putrescine and acetylated polyamines. Figure created using BioRender.com.

Polyamine transport is crucial for regulating polyamine content. However, the precise molecular mechanisms involved remain inadequately understood. As illustrated in **Figure 2**, two primary models exist regarding polyamine transport. The first model posits that polyamines first enter the cytoplasm through membrane permeases before moving into acidic vesicles via transporter proteins. This process relies on V-ATPase activity and an outward proton gradient (5). The vesicular polyamine transporter protein (VPAT), encoded by SLC18B1, plays a pivotal role in storing spermine and spermidine in vesicles and releasing them from secretory cells (6). The second model suggests that polyamines enter cells via endocytosis by caveolin and glypican. Within endosomes, polyamines are released through a nitric oxide(NO)-mediated oxidative mechanism and exported via polyamine transporter proteins (7). ATP13A2 is primarily responsible for exporting spermine, N1-acetylspermine, and spermidine, while putrescine and acetylated polyamines (AcPolyamines) generated by spermine/spermidine N-acetyltransferase (SAT1) are exported through SLC3A2 (8, 9). Notably, both transport mechanisms are supported by biochemical evidence and likely operate concurrently in polyamine transport.

Recent advancements in research techniques have led to increased focus on P5B-type transporter ATPases and solute carrier transporter proteins (SLCs). However, only a limited number of transporter proteins have been biochemically or structurally validated, with ATP13A2’s function becoming clearer. ATP13A2 is an endo/lysosomal transporter protein (10) that selectively filters polyamines by binding and autophosphorylating ATP, thus forming a narrow channel within the lysosomal lumen. Upon the release of the phosphate group, it transitions into a ‘pump-channel intermediate’, facilitating the transport of polyamines from the lysosome to the cytoplasm (11). Jianqiang Mu et al. have analyzed the high-resolution structures of ATP13A2 in six intermediate states using single-particle cryo-electron microscopy, integrating biochemical experiments and molecular dynamics simulations to elucidate its conformational cycling (12). Additionally, Marine Houdou et al. demonstrated that ATP13A3 uptakes polyamines more rapidly and efficiently than ATP13A2 using a novel green fluorescent polyamine (13). The domain of polyamine transport system (PTS) is known as ‘black box’. However, propelled by the advancement of techniques, more biochemical and structural properties regarding polyamine transporters will be unveiled.

#### 2.1.3 Catabolism

Spermines can be catabolized into spermidines either through a one-step enzymatic reaction mediated by spermine oxidase (SMOX) or via a two-step reaction involving spermine/spermidine N1-acetyltransferase (SSAT) coupled with the peroxisomal enzyme N1-acetylpolyamine oxidase (APAO). SSAT transfers an acetyl group from acetyl coenzyme A to the N1 position of either spermidine or spermine. The acetylated polyamines can either be excreted from the cell through polyamine transport proteins or converted back into spermidine or spermine by polyamine oxidase (PAOX). Both PAOX and SMOX can generate substantial amounts of reactive oxygen species (ROS) and toxic by-products during polyamine catabolism, including 3-amino-propionaldehyde (3-AP), 3-acetamido-propionaldehyde (3-AAP), and hydrogen peroxide (H₂O₂), leading to oxidative damage (14). Acrolein, a toxic byproduct, can be produced from 3-AP (15). As for the putrescine, it can be oxidized by diamine oxidase (DAO) to produce 4-aminobutyraldehyde and H_2_O_2_. Subsequently, aldehyde dehydrogenase (ADH) can facilitate 4-aminobutyraldehyde into gamma-aminobutyric acid (GABA), a crucial neurotransmitter (16).

It’s noteworthy that spermidine is the sole substrate for hypusination of eukaryotic initiation factor 5A (eIF5A) through 2 enzymatic steps. The precursor form of eIF5A undergoes modification through the attachment of the 4-aminobutyl group of spermidine by deoxyhypusine synthase (DHPS) to a particular lysine residue in the eIF5A precursor protein. Subsequently, deoxyhypusine hydroxylase (DOHH) transforms deoxyhypusine into hypusine, thereby activating eIF5A (17). What’s more, eIF5A1 and eIF5A2 are known as critical players in the regulation of translation and cellular metabolism, especially in cancer biology. eIF5A1 play a dual role in tumor progression (18). While eIF5A2 is known as oncogene, more directly associated with oncogenic processes, including invasion, metastasis, and drug resistance in cancer. However, the specific mechanism remains unclear. Some studies suggest that eIF5A2 facilitates cancer cell protrusions and invasive capacity by activating the translation of key proteins (19). Some indicate that eIF5A2 increases metastasis and angiogenesis by promoting the signaling pathway associated with HIF1a (20). Moreover, hypusinated eIF5A promotes tumor growth by up-regulating MYC elongation (21). Both isoforms are characterized by the unique post-translational modification known as hypusination, which is critical for their biological functions.

### 2.2 Polyamine metabolism in cancer tissues

Many types of malignant tumors exhibit dysregulation of polyamine metabolism, characterized by the accumulation of elevated polyamine levels in cancerous tissues.

#### 2.2.1 Upregulation of enzymes and polyamine transport systems

Early research indicated that polyamine metabolism in cancer tissues shares similarities with that in normal tissues. However, the expression of mediating enzymes is altered. In colorectal cancer tissues, polyamine levels, along with the activities of ODC and AMD1, were found to be three to four times higher than in normal colon tissues (22). The upregulation of ODC may be related to the unique characteristics of the tumor microenvironment. Hypoxic conditions increase ODC expression and enhance polyamine synthesis through the action of hypoxia-inducible factor 1-α (HIF-1α), supporting rapid tumor cell proliferation (23). This phenomenon may also correlate with the dysregulation of oncogenes, including MYC, JUN, FOS, KRAS, and BRAF (24). Among these, MYC is particularly noteworthy, as aberrant c-MYC expression is observed in approximately 70% of human cancers (25). The rate-limiting enzymes of polyamine metabolism, ODC and AMD1, are direct transcriptional targets of MYC (26). MYC enhances polyamine levels by increasing the expression of polyamine-synthesizing enzymes (27).

Similarly, catabolic enzymes are often upregulated in cancerous tissues, resulting in decreased levels of spermine and spermidine, which may promote chronic inflammation and contribute to cancer progression (28). Additionally, reactive oxygen species induced by catabolic oxidation can induce apoptosis in epithelial cells and increase DNA damage, heightening the risk of tumorigenesis (29). The expression and protein content of SSAT mRNA are elevated approximately 5-10-fold in primary tumor tissues of the prostate, breast, and lung compared to adjacent normal tissues (30). SSAT can serve as a tumor marker, and its activation is associated with various factors, including oncogenes and cancerous tissues. Divya Murthy et al. found that MUC1, a mucin overexpressed in pancreatic cancer, regulates polyamine catabolism by stabilizing HIF-1α expression, enhancing its occupancy of the SAT1 promoter, and activating SAT1 gene expression, thereby promoting polyamine metabolism and the production of N1-N-acetylspermine and N8-N-acetylspermine (31). Ling Deng et al. discovered that in triple-negative breast cancer (TNBC), MYC promotes the translation of U2 SURP through a eIF3D-dependent mechanism, facilitating the selective splicing of spermine/spermidine N1-acetyltransferase 1 (SAT1) precursor mRNA by removing intron 3. This process leads to increased stability of SAT1 mRNA and enhanced protein expression levels (32). Similarly, the expression of SMOX is upregulated in gastric, lung, breast, prostate, and colorectal cancers, which may serve as a prognostic indicator for colon cancer (33). However, it has been suggested that genetically determined SMOX activity does not correlate with the risk of childhood neuroblastoma or adult cancers, including gastric, lung, breast, prostate, and colorectal cancers (34).

Another characteristic of malignant tumors is the upregulation of PTS. However, few studies have distinctly identified the transporter proteins responsible for increasing polyamine levels in cancer cells. Recently, Sarah van Veen et al. demonstrated that ATP13A4, a member of the P5B-ATPase family, contributes to enhanced polyamine uptake in breast cancer cells, with no apparent role in normal tissues. This finding was established by comparing the normal cell line MCF10A with the breast cancer cell line MCF7. ATP13A5 and ATP13A4 are closely related isoforms that may also be involved in polyamine transport (35). Vandana Sekhar et al. noted that in breast cancer cells, SLC12A8 is incapable of transporting polyamines, while ATP13A3 plays a crucial role in polyamine transport in cells deficient in spermine and spermidine. ATP13A3 may accumulate exogenous spermine at the membrane when cells lack polyamines. Upon spermine uptake, ATP13A3 is internalized into vesicles that facilitate the transport of spermine within the cell (36).

In general, there is much evidence indicating that polyamines are upregulated by disordered enzymes and PTS. Yet, the mechanism of polyamine elevation is manifold and intricate. Meanwhile, more research is required to verify specific transporter involved in polyamine transportation in cancers.

#### 2.2.2 *De novo* polyamine synthesis in cancer

Recent studies have identified a novel pathway for the *de novo* synthesis of polyamines in cancer. Glutamine, a minor precursor of polyamines, can even substitute arginine as the raw material for *de novo* polyamine synthesis in tumors. Min-Sik Lee et al. demonstrated that in pancreatic ductal adenocarcinoma (PDAC), glutamine serves as the major carbon donor for polyamine synthesis, with this synthetic pathway being tissue-specific. In this pathway, glutamine is converted to ornithine via a two-step reaction catalyzed by pyrroline-5-carboxylate synthase (P5CS) and ornithine aminotransferase (OAT). Their findings showed that pancreatic tumors were enriched with large amounts of labeled ornithine and putrescine through isotopic tracing, a pattern not observed in the normal pancreas (37). This synthetic pathway is mediated by OAT and polyamine synthase and can be induced by activated KRAS (38). But it is yet unclear if the novel mechanism holds true for other types of cancer.

## 3 Polyamines drive tumorigenesis

**Figure 3:**
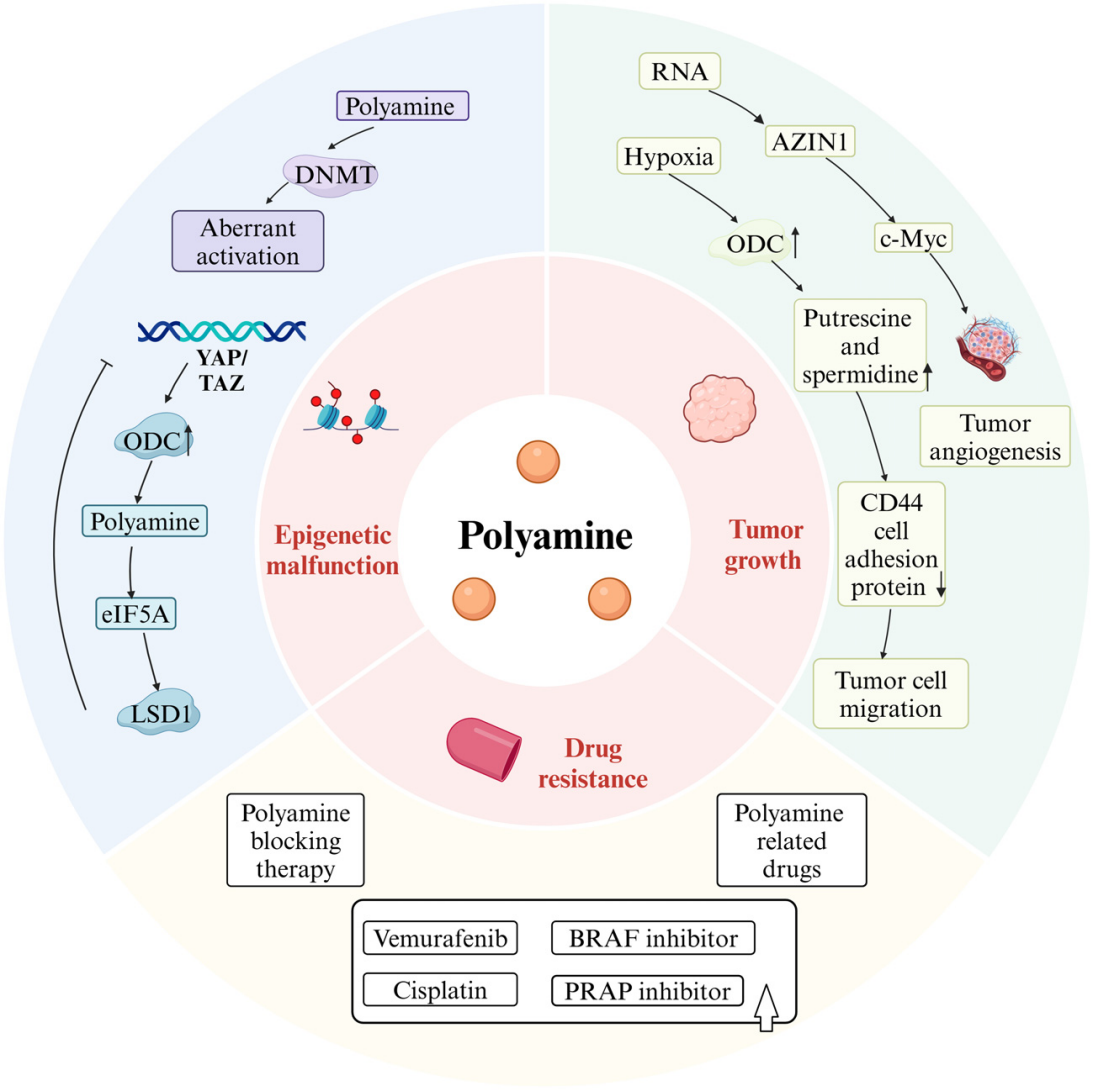
Polyamines drive tumorigenesis. Figure created using BioRender.com.

Given that polyamines are upregulated in tumor tissues, it is no surprise that elevated polyamine levels augment the invasiveness and metastatic potential of cancer cells. (as shown in **Figure 3**).

### 3.1 Polyamine metabolism promotes aberrant epigenetic modification

Altered polyamine metabolism affects epigenetic modifications in tumor cells, contributing to their phenotypic transformation. In cells where polyamine synthesis is inhibited, the activity of AMD1 is elevated, leading to a reduced concentration of SAM, the major donor of DNA methyl groups, and an increased concentration of decarboxylated SAM (dcSAM), a potent inhibitor of DNA methyltransferase (DNMT) (as shown in **Figures 1**, **3**). While polyamine deprivation does not alter the protein levels of DNMT, it regulates methylation levels by activating DNMT1, 3A, and 3B, resulting in a genome-wide aberrant methylation state (39), where methylation is promoted in certain regions and suppressed in others (40). Changes in methylation status can modulate chromatin accessibility, triggering aberrant gene transcriptional activity and genomic instability, which may drive tumorigenesis. Additionally, oncogenes can influence epigenetic modifications by regulating key metabolic pathway enzymes. Hongde Li et al. revealed the YAP/TAZ-polyamine-eIF5A-Hyp-LSD1 axis, linking polyamine-mediated eIF5A hypusination to oncogenic signaling and epigenetic remodeling (41).

There is evidence that polyamines play a vital part in epigenetic modification of normal tissues, but the mechanism in cancer tissues needs to be explored.

### 3.2 Polyamines promote tumor growth and metastasis

Polyamine levels are elevated in many human cancers (42), serving as metabolites that regulate numerous cellular processes (43). Polyamines are recognized as overexpressed tumor biomarkers in several cancer types, including breast and prostate cancers, Polyamines are recognized as overexpressed tumor biomarkers in several cancer types, including breast and prostate cancers, and their levels tend to increase with the grade of malignancy (44). Under hypoxia conditions, ODC induces and stimulates the uptake of exogenous polyamines and the biosynthesis of intracellular polyamines, resulting in elevated levels of putrescine and spermidine. After HT-29 colon cancer cells were exposed to spermine, hypoxia-induced CD44 cell adhesion protein expression was further reduced, and tumor cell migration, invasion and metastasis were promoted. Consumption of polyamines with DFMO during hypoxia increases apoptosis in multiple cancer cell lines, suggesting that hypoxic tumor cells have increased demand for polyamines (24). Furthermore, it has been shown that antizyme inhibitors (AZIN1), which promote production of polyamines, increase IL-8 by postponing c-Myc degradation via the OAZ2-mediated ubiquitin independent proteasome pathway (45), thus promoting tumor angiogenesis. The increased rates of RNA-edited AZIN1 in human malignancies are associated with increased tumor angiogenesis, improved cancer cell stemness, and increased cancer cell proliferation (46). Additionally, putrescine, spermidine, and spermine are critical metabolites in mammalian tissues, playing significant roles in cell cycle regulation, with brain tumor growth often relying entirely on exogenous spermidine supply (47). Increased polyamine levels in brain tumors correlate with heightened ODC activity. Upregulation of ODC is evident in all astrocytomas, ventricular meningiomas, and meningiomas compared to non-tumorigenic brain tissues, increasing with malignancy grade (48).

To summarize, polyamine-mediated hypoxia, angiogenesis, and cancer cell stemness and cell cycle dynamics all result in cancer cells development and metastasis, implying a potential target for anticancer therapies.

### 3.3 Polyamines enhance tumor resistance to therapeutic drugs

Drug resistance in certain tumors complicates treatment strategies. Recent studies suggest a positive association between polyamines and drug resistance in tumors. For instance, Byung-Sun Park et al. demonstrated that AMD1, a key enzyme in A375 melanoma responsible for vemurafenib resistance, upregulates polyamine synthesis, thereby increasing mitochondrial activity and promoting resistance (49) Vemurafenib is commonly used to treat melanoma with BRAF V600E mutations, but tumor resistance diminishes its therapeutic efficacy. Inhibiting polyamine synthesis has been shown to help overcome vemurafenib resistance in melanoma (49).

Additionally, therapies targeting SAT1 can help overcome chemotherapy resistance in patients with PDAC (31). Aberrant expression or stimulation of SAT1 can restore cisplatin sensitivity in cell culture systems (50). α-Difluoromethylornithine (DFMO) has been shown to reduce resistance to poly(ADP-ribose) polymerase (PARP) inhibitors in ovarian cancer, acting as an effective adjuvant chemotherapy (51). In TNBC, known for its resistance to chemotherapy and poor prognosis, existing studies indicate that inhibiting polyamine synthesis improves chemotherapy sensitivity (52). Collectively, these studies suggest that inhibiting polyamine synthesis and metabolism may help address therapeutic challenges in drug-resistant tumors, providing new hope for treatment. Furthermore, certain polyamine-related drugs can also mitigate tumor drug resistance; for example, the combination of a novel arylmethyl-polyamine (AP) with the BRAF inhibitor (PLX4720) significantly delays recurrence in PLX4720-resistant melanoma (53). Additionally, homospermidineplatin (4a) effectively inhibits *in situ* tumor growth and reverses cisplatin resistance (54).

## 4 Polyamines link cancer cells and tumor immune microenvironment

**Figure 4:**
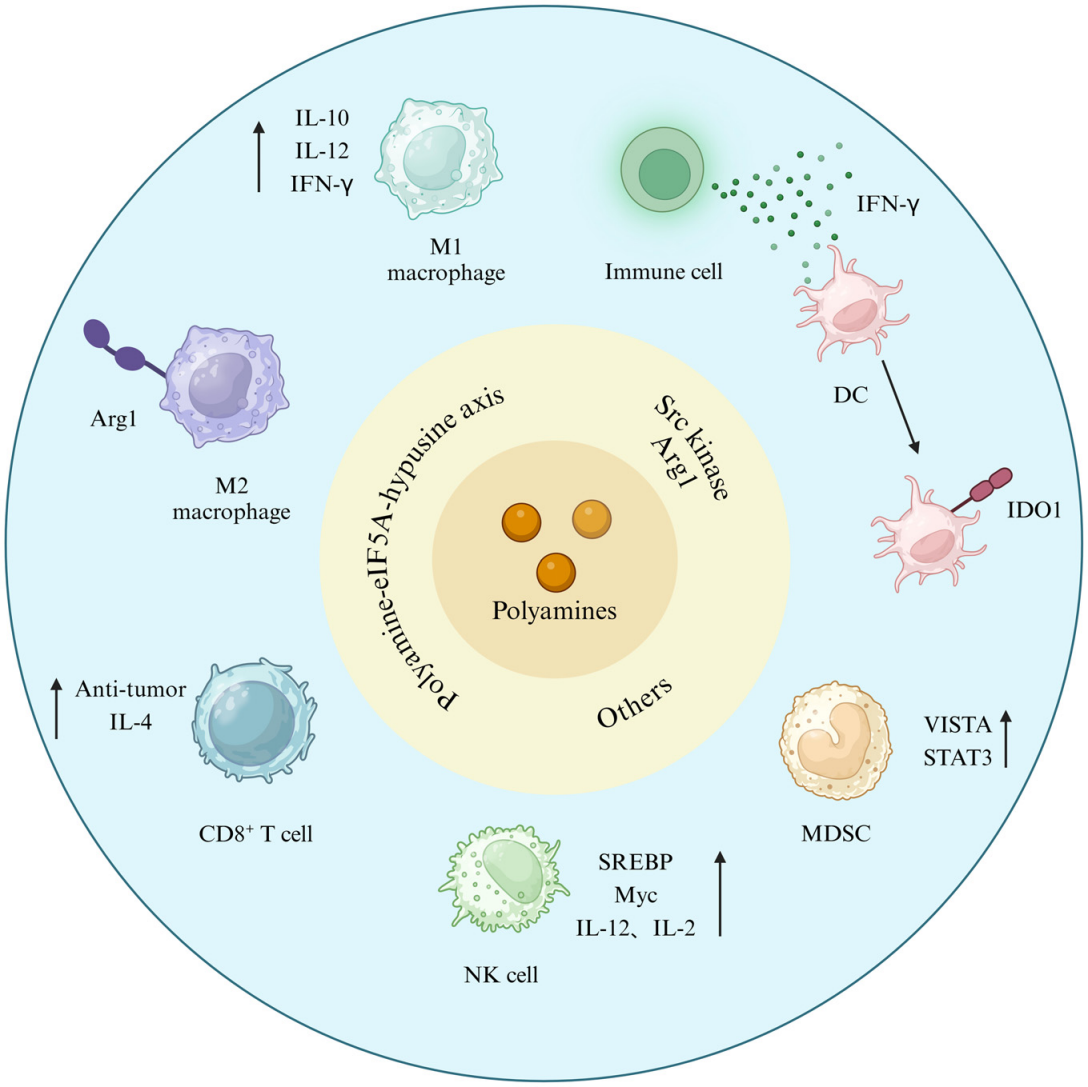
Polyamines link cancer cells and TIME. Figure created using BioRender.com.

As illustrated in **Figure 4**, polyamine metabolism exerts diverse immunomodulatory effects. Animal experiments have shown that polyamine deprivation prevents the development of tumor-induced immunosuppression (44).

### 4.1 Polyamines modulate T cell differentiation and inhibit anti-tumor immunity

There is a unique post-translational modification called hypusination, which occurs on a specific lysine residues of a known protein eIF5a and the hypusinated effects of polyamine-dependent eIF5a have been shown to be important in controlling T cell metabolism and function. Spermidine has been demonstrated to activate autophagy in cardiomyocytes, neurons, satellite cells, CD8^+^ T cells, and B cells (55). eIF5a hypusination declines as the body ages (56), nevertheless, it can augment the anti-tumor efficacy of CD8^+^ T cells lymphocytes by directly targeting mitochondrial trifunctional protein (MTP) (57), and can be reinstated with dietary supplementation with spermidine (58). Numerous results indicate that spermidine prolongs lifespan across species in an autophagy-dependent manner and mitigates cancer and age-related illnesses, such as cardiovascular disease and dementia (55, 59, 60). Spermidine, however, is notable for its regulation of CD4^+^ T cell development *in vitro*, preferring directing early T cells towards a regulatory phenotype. Following spermidine treatment, activated T cells deficient in the autophagy gene Atg5 exhibited a diminished capacity to upregulate Foxp3 compared to wild-type cells. The findings indicate that the polarizing impact of spermidine necessitates a functional autophagic system (61). Furthermore, tumor cells generated endogenous spermidine, which reduced plasma membrane cholesterol levels, consequently reducing CD8^+^ T cell antigen receptor aggregation and obstructing T cell activation (62). Furthermore, putrescine obstructs the development of cytolytic T lymphocytes (CTLs), thereby undermining anti-tumor immunity (59).

In summary, the hypusinated effects of polyamine-dependent eIF5A can enhance the anti-tumor effects of CD8^+^ cells directly via MTP and can be restored by dietary supplementation. In addition, tumor cells can release endogenous spermidine, which blocks T cell activation and thus suppresses anti-tumor immunity. Whether these mechanisms are consistent in different requires more research to verify.

### 4.2 Polyamines modulate the cytotoxicity of NK cells

The SREBP-cMyc axis is essential for the *de novo* production of polyamines, which subsequently facilitates NK cell metabolism and functionality (63). SREBP and cMyc are two significant metabolic regulators of NK cells.

Myc is a crucial regulator of immune cell activity and function (27), the rate-limiting enzyme in polyamine production. During the progression of colon cancer, compromised energy production in tumor cells facilitates the consumption of NK cell polyamines by these cells, which inhibits cMyc in NK cells and disrupts their glycolysis, ultimately diminishing their cytotoxic ability (64). The findings indicated that NK cells may counteract the inhibitory effects of cMyc and the disruption of glycolytic energy supply, thereby reinstating the cytotoxic activity of NK cells following the administration of a specific amount of spermidine. The findings indicate that cMyc-regulated polyamine levels and glycolytic availability are essential for the immunological function of NK cells (64). In colon cancer, the administration of exogenous polyamines can enhance tumor proliferation and glycolysis, fulfill the tumor’s requirements, and prevent the invasion of the intestinal mucosa by tumor consumption of polyamines (64). In RPMI-8402 cells, DMSO prompted a cMyc-dependent reduction in ODC activity and consequent depletion of intracellular polyamine levels, correlating with programmed cell death and cell growth arrest (65).

The sterol regulatory element binding protein (SREBP) is another regulator that modulates cMyc expression via cytokines IL-2 and IL-12, therefore facilitating the *de novo* synthesis of polyamines (63). The induction of SREBP target genes relies on the mammalian target of rapamycin complex 1 (mTORC1), and SREBP activation is heightened in cytokine-stimulated NK cells. Furthermore, it has been demonstrated that mTORC1 inhibition does not influence cMyc expression in NK cells activated by IL-2 and IL-12 (66). Experimental data indicated that supplementing NK cells with impaired SREBP activity with exogenous polyamines only partially restored NK cell function, implying that additional SREBP-regulated processes or endogenously synthesized polyamines are crucial for enhancing NK cell effector function (63).

Spermine catalyzes the substrate for eIF5a via the sequential actions of the enzymes deoxyhydroxylase (DHPS) and deoxyhydroxylase (DOHH), with all proteins in this pathway expressed in cytokine-activated NK cells, which regulate the *de novo* synthesis of polyamines to supply substrates for catalepsy.

As mentioned above, SREBP and cMyc are two significant metabolic regulators of NK cells. The induction of SREBP is dependent on mTORC1, and SREBP activation is increased under cytokine stimulation, and cMyc expression is inhibited, but inhibition of mTOR1 does not affect cMyc expression in NK cells. In addition, the depredation of polyamines inhibited cMyc, thus reducing the killing activity of NK cells, and supplementing exogenous polyamines could restore its function to a certain extent.

### 4.3 Polyamines promote macrophage M2 polarization and inhibits its immunological activity

Polyamines are significant immunomodulators that influence macrophage polarization and are crucial in tumorigenesis. Recent studies indicate that polyamines can facilitate the alternative activation of OXPHOS-dependent macrophages, suppress the classical activation of aerobic glycolysis-dependent macrophages, and polarize macrophages towards the M2 phenotype via polyamine (4). Spermidine serves as a substrate for the production of conserved lysine residues in polyamine-eIF5A-hypusine axis. The hypusinated form of eIF5A (eIF5A^H^) enhances the effective production of specific mitochondrial proteins associated with the tricarboxylic acid (TCA) cycle and oxidative phosphorylation (OXPHOS) (67). Furthermore, it has been shown that spermine-induced M2 polarization is facilitated by mitochondrial reactive oxygen species (mtROS). mtROS activates AMP-activated protein kinase (AMPK), which induces the upregulation of HIF-1α and autophagy, essential for the production of M2-associated genes such as Arg1 and Chil3 (68). Nakamura et al. discovered that exogenous putrescine prompted metabolic reprogramming similar to IL-4 activation (69).

Polyamines can potentially influence macrophage functions, including pro-inflammatory and anti-inflammatory actions. Spermine efficiently enhances interleukin-10 (IL-10) production in macrophages while suppressing the synthesis of pro-inflammatory cytokines such as tumor necrosis factor (TNF), IL-1, IL-12, IL-6, IFN-γ, and MIP-1β (70). Similarly, spermidine selectively inhibits the secretion of TNF-α, IL-1β, IL-12, and chemokine monocyte chemoattractant protein-1(MCP-1) by macrophages, directly downregulates NOD-like receptor protein 3 (NLRP3) inflammasome activation in these cells, and lowers the co-stimulatory molecules CD80 and CD86 to suppress M1-type macrophages (71). Furthermore, the impact of polyamines on cytokines is intricately associated with the concentration and classification of polyamines (72). Putrescine inhibited macrophage polarization to the M1 phenotype by down-regulating IL-8 (73).

Taken together, polyamines participate in regulating the differentiation process of macrophages and thereby facilitate the establishment of an immunosuppressive tumor microenvironment.

### 4.4 Polyamines modulate other immune cell activities

MDSC-induced L-arginine depletion decreased CD 3^+^ expression in activated T cells and suppressed antigen-specific proliferation of OT-1 and OT-2 cells (74). Numerous investigations have shown that polyamines activate myeloid-derived suppressor cells in an inhibitory manner and facilitate their metabolism. The V-domain suppressor of T cell activation (VISTA)/STAT3/polyamine axis recently regulates the development of myeloid cells into MDSC.VISTA triggers STAT3, which facilitates MDSC development through the initiation of a transcriptional and metabolic pathway (75).

In addition, research has shown that polyamines can be synthesized by dendritic cells or by Arg1^+^ myeloid-derived suppressor cells. They govern the conversion of dendritic cells into an indoleamine 2,3-dioxygenase 1(IDO1)-dependent immunosuppressive state by activating Src kinases that possess IDO1 phosphorylation activity (76). Gervais et al. discovered that the incorporation of putrescine into the milieu of dendritic cells inhibited their capacity to effectively cross-present exogenous antigens, leading to diminished immunological functionality of dendritic cells (77).

### 4.5 Polyamines regulate cytokines

Cytokine regulation is influenced by polyamine metabolism. Spermine has been demonstrated to act as a metabolic inhibitor of Janus kinase (JAK) signaling. Spermine directly engages with the N-terminal structural domain of JAK1, disrupting the interaction between JAK1 and cytokine receptors, which consequently inhibits JAK1 phosphorylation induced by cytokines IFN-I, IFN-II, IL-2, and IL-6 (78). Additionally, it does not influence the membrane localization or mRNA expression of interferon α/β receptor (IFNAR)1 and IFNAR2 in target cells.

Moreover, polyamines participate in the inflammatory response via SMOX, functioning as either a positive or negative regulator of inflammatory variables contingent upon the unique tissue environment and the method of polyamine replenishment (79). Polyamines can enhance the expression of inflammatory factors under specific conditions. Following infection with Citrobacter rodentium, levels of the chemokines CCL 2, CCL 3, CCL 4, CXCL 1, CXCL 2, and CXCL 10, together with the cytokines IL 6, TNF α, CSF 3, IFN γ, and IL 17, were significantly raised in the colon. All of these factors were down-regulated in mice deficient in Smox. Conversely, in the dextran sodium sulfate(DSS)-induced colitis paradigm, the concentrations of IL-6, CSF-3, and IL-17 were significantly increased in mice deficient in Smox (80). In a colitis-associated cancer (CAC) model treated with oxidized azomethine (AOM)-DSS, SMOX deficiency correlated with exacerbation of CAC, heightened production of α-defensins, and supplementation with spermidine ameliorated these phenotypes, functioning as a preventive agent in CAC (81). Inflammatory factors are chemotactic and facilitate the function of immune cells in suppressing the growth and migration of cancer cells (82). A significant characteristic of the tumor microenvironment is persistent inflammation, and research on polyamines and inflammation may elucidate a survival strategy for tumor cells to circumvent the immune response.

## 5 Polyamines-driven targeted anti-tumor therapy

### 5.1 Rigorous regulation of polyamine biosynthesis, transporting and catabolic enzymes

Polyamines are crucial for cellular proliferation, and neoplasms necessitate elevated levels of polyamines, which promote angiogenesis, augment biomass through enhanced protein translation, facilitate the survival and metastasis of epithelial tumor cells, influence the production, viability, and functionality of tumor-infiltrating immune cell subsets, and suppress organismal immunity, thereby aiding tumors in evading immune responses. Consequently, polyamine metabolism, transport, and catabolism are often dysregulated in cancer (83). Stringent regulation of enzymes implicated in polyamine production, transport, and catabolism can impede tumor proliferation. Inhibitors of nearly all enzymes involved in polyamine synthesis, transport, and catabolism have been identified to date (as illustrated in **Table 1**).

**Table 1:** Inhibitors of polyamine synthesis, transport, and catabolic enzymes.

Drug	Target	Structure	Status
DFMO	ODC	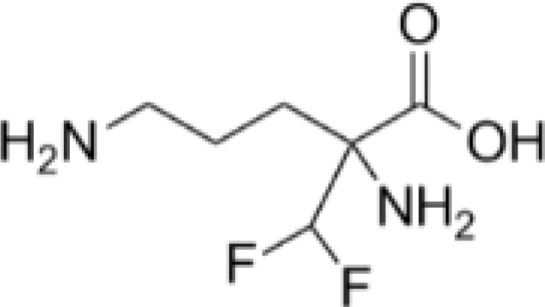	Broadest range of irreversible inhibitors
SAM486A	AdoMetDC	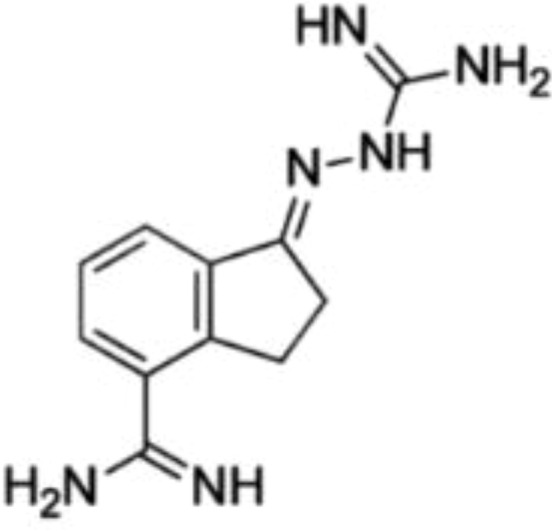	Anti-tumor effects but severe toxicity
indomethacin	SSAT-1	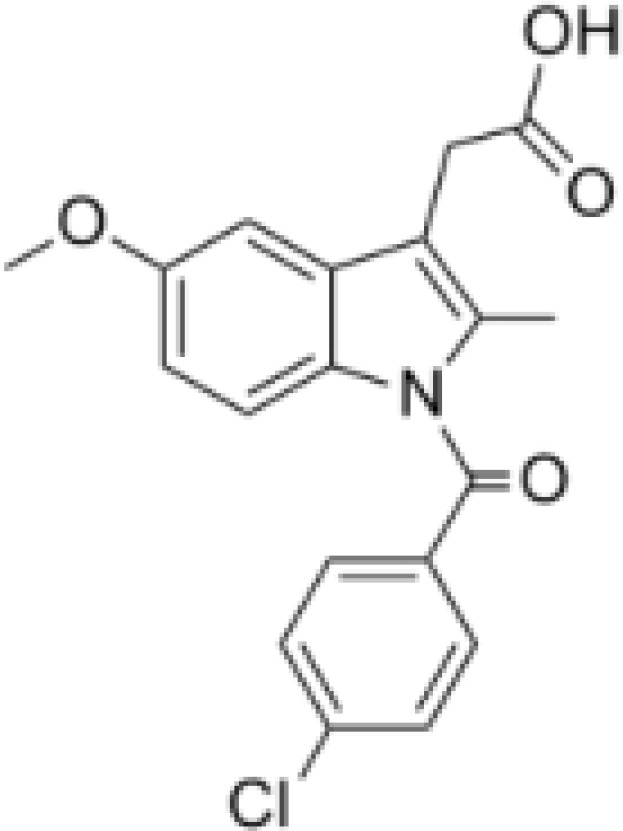	Not yet in clinical use
sulindac	SSAT-1	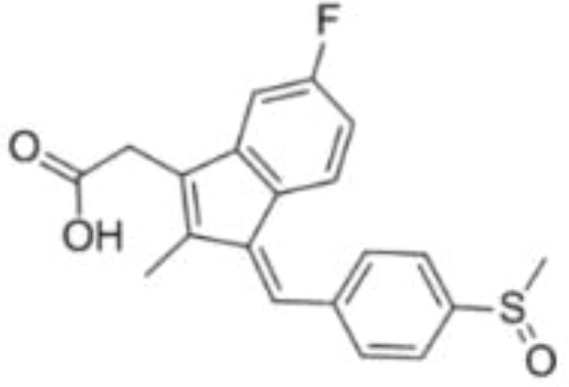	Not yet in clinical use
17β-Estradiol	PAOX	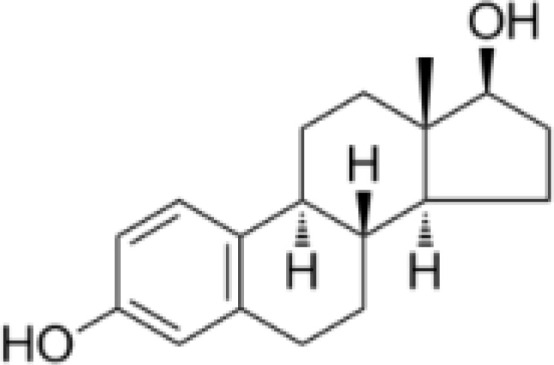	Not yet in clinical use
MDL 72527	SMOX and/or PAOX	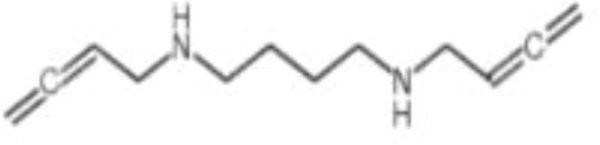	Not yet in clinical use
AMXT 1501	polyamine transport	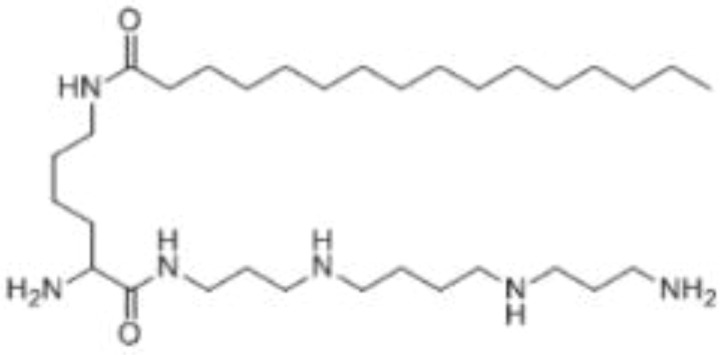	Poor clinical outcomes with single drug use
MTA	PRMT5	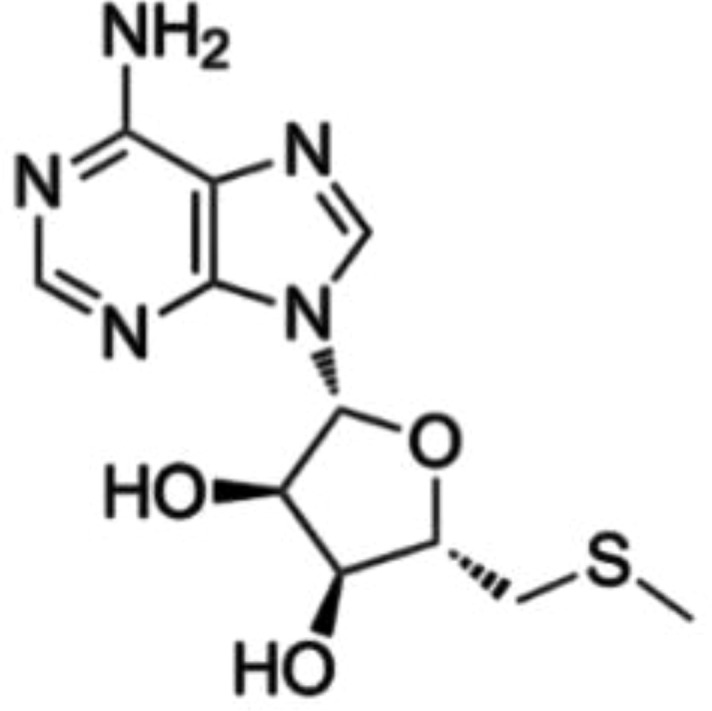	Not yet in clinical use
BENSpm	ODC etc.	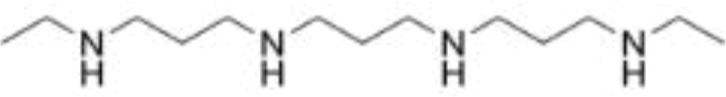	CNS toxicity, poor clinical outcome
PG-11047	ODC etc.	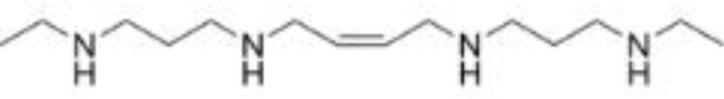	Often well-tolerated in clinical trials, stabilizes cancer disease

DFMO, α-Difluoromethylornithine; ODC, ornithine decarboxylase; SAM486A, 4-amidinoindan-1-one 2′-amidinohydrazone; AdoMetDC, S-adenosylmethionine decarboxylase; SSAT-1, spermidine/spermine-N1-acetyltransferase-1; PAOX, polyamine oxidase; MDL 72527, N1,N4-di(buta-2,3-dien-1-yl)butane-1,4-diamine; AMXT 1501, an orally active inhibitor of the polyamine transport system; MTA, 5-methylthioadenosine; PRMT5, protein arginine N-methyltransferase 5; BENSpm, N1,N11-bis(ethyl)nordemethylspermine; PG-11047, N1,N12-bis(ethyl)-cis-dehydrogenated spermine.

Ornithine decarboxylase is the principal rate-limiting enzyme in polyamine production, and DFMO is the most prevalent irreversible inhibitor of ODC. Numerous experimental studies have demonstrated that DFMO possesses oncostatic properties. DFMO can reduce neuroblastoma at clinically significant levels by targeting cells critical for neuroblastoma carcinogenesis (84). DFMO can inhibit PDAC via suppressing MYC (85). DFMO is presently FDA-approved for the treatment of African sleeping sickness; nevertheless, it has not yet achieved widespread recognition for cancer therapy. DFMO is associated with toxicity; for instance, it may induce hearing loss even at low dosages (86). Moreover, DFMO diminishes intracellular polyamines and suppresses tumor cell proliferation across several model systems, yet it does not demonstrate substantial anticancer efficacy (87). Furthermore, many innovative polyamine synthesis inhibitors, like clofazimine (CLF), also impede multiple myeloma through the aryl hydrocarbon receptor/polyamine biosynthesis axis (88). Drugs such as AMXT1501 and Trimer44NMe inhibit tumor growth and diffusion by inhibiting polyamine transport. In conclusion, as an effective polyamine synthesis blocker, DFMO shows certain potential for cancer treatment, but the accompanying toxicity and the body’s compensatory mechanisms limit its effectiveness in tumor treatment.

### 5.2 Combination therapy utilizing polyamine inhibitors synergizes with other pharmacological agents

Single DFMO agents exhibit restricted therapeutic efficacy in clinical tumors, and DFMO-mediated inhibition of ODC induces a compensatory mechanism whereby tumor cells augment the uptake of exogenous polyamines, including those present in the tumor microenvironment, dietary metabolites, microbiota, and particularly from decidualized or damaged cells in the intestinal lumen (24). Tracy Murray Stewart et al. established that the suppression of cell proliferation due to DFMO-induced polyamine depletion was mitigated by exogenous N8-acetylspermidine (at physiological concentrations), which was metabolized to spermine exclusively in cell lines exhibiting histone deacetylase-10 (HDAC10) activity (89). Combination medicines have demonstrated superior efficacy relative to monotherapy. For example, DFMO increases the anti-tumor activity of PARP inhibitors in the treatment of ovarian cancer^4^.

#### 5.2.1 Combination therapy with polyamine transport inhibitor and difluoromethylornithine

Polyamine blockade therapy (PBT) is an amalgamation of difluoromethylornithine (DFMO) and an innovative polyamine transport inhibitor (PTI), specifically a trimeric polyamine molecule, utilized in oncological treatment. Following simultaneous administration of DFMO and trimeric PTI, autophagic flux was markedly diminished in tumor-infiltrating M2-like macrophages and myeloid-derived suppressor cells (MDSC) (83). The antitumor efficacy of PBT aligns with experiments indicating that its oncostatic effects may be associated with the direct targeting of tumor-promoting Ly6G+ myeloid cells, the suppression of M2-like tumor-associated macrophage polarization, and its targeted removal of tumor-infiltrating and immunosuppressive myeloid populations (75) AMXT 1501 is the most extensively researched PTI. AMXT 1501 was engineered as a polyamine mimetic with a lysine-spermine framework with a C16 lipophilic substituent on the epsilon-nitrogen atom of the lysine segment to enhance its capacity to inhibit cellular uptake of spermine in the nanomolar range while remaining impermeable to the cellular membrane. AMXT 1501 obstructs polyamine transporter proteins at the plasma membrane and is not absorbed by the cell (90). The synergistic therapy utilizing DFMO and AMXT 1501 depends on T-cells to eradicate polyamines from tumors, thereby suppressing tumor proliferation. Recent investigations indicate that the combination of Loratinib and AMXT-1501 inhibits anaplastic lymphoma kinase (ALK), subsequently inhibiting SLC3A2 and thereby obstructing cell proliferation in neuroblastoma cell lines (91). Administration of DFMO and AMXT 1501 before the surgical excision of the initial tumor induces protective immunity against tumor recurrence (92). AMXT 1501 can surmount tumor resistance to DFMO and diminish tumor cell viability following DFMO-mediated suppression of ODC. The combination of AMXT-1501 and DFMO for solid tumors is presently undergoing phase 1 clinical studies. Combination therapy involving AMXT1501 and DFMO presents hazards, including gastrointestinal disorders (diarrhea, nausea, vomiting, abdominal discomfort, dyspepsia, and fecal discolouration) and systemic disorders (fatigue and fever) (93). A limited cohort of patients experiences thrombocytopenia, predominantly attributable to DFMO (93). Furthermore, Trimer44NMe is utilized to obstruct polyamine transport (94). Aiste Dobrovolskaite et al. utilized pancreatic ductal adenocarcinoma L3.6pl cells, characterized by elevated polyamine transport activity, to demonstrate that polyamine lassos constructed from nine- and twelve-membered triazacyclic macrocycles, attached to one end of homotrimethylene tetracarboxamidine, as well as smaller triazacyclononane-based lasso, exhibited comparable efficacy as inhibitors of polyamine transport, effectively obstructing the uptake of ^3^H-spermidine. The smaller lasso is better to the larger lasso (95).

PTI reduces polyamine levels by obstructing polyamine transport, transforming cold tumors into hot tumors and enhancing the immunogenicity of cold tumors (24). GCN2 signaling in stromal cells, which is dependent on polyamines, facilitates tumor growth and fosters an immunosuppressive tumor microenvironment; also, the anticancer effects of PBT are partially mediated through the targeting of GCN2 (96). Simultaneously, PBT has synergistic anticancer efficacy with PD-1 blockage, which mitigates the suppression of cytotoxic T-cell activity by obstructing PDL1/PD-1 signaling, leading to tumor growth inhibition and enhanced survival rates.

#### 5.2.2 Concurrent Azacytidine and difluoromethylornithine therapy

Azacytidine (AZA) has extensive antimetabolic action and impedes DNA methylation when incorporated into DNA (97). AZA is licensed by the FDA for the chemotherapy of acute myeloid leukemia and for the treatment of individuals with myelodysplastic syndromes in pre-leukemic hematological illnesses (98). Research indicates that the combination of AZA and DFMO results in reduced levels of putrescine and spermine in tumor cells, as well as a diminished total count of macrophages inside the tumor microenvironment, accompanied by an increased ratio of M1 to M2 macrophages. M1 macrophages exhibit anti-tumor properties, whereas M2 macrophages facilitate tumor progression. Subsequent to the combination therapy, the tumor microenvironment exhibited a notable augmentation in T cells, NK cells, and IFNγ+ lymphocyte populations (87). This signifies that the combined therapy of AZA and DFMO exhibits anti-tumor properties.

### 5.3 Utilization of polyamine analogs in lieu of polyamine synthase inhibitors

The inhibition of polyamine synthesis activates a compensatory mechanism in the body, rendering direct inhibition of polyamine synthase minimally effective in treating advanced cancers. In contrast, polyamine analogs can effectively disrupt this compensatory mechanism by promoting the catabolism of polyamines without substituting the growth support function of natural polyamines. Intracellular accumulation of polyamine analogs selectively enhances the activities of SSAT and SMOX, hence promoting polyamine catabolism and reducing their production (42). Numerous symmetrically substituted polyamine analogs demonstrate regulation of polyamine metabolism *in vitro* and show efficacy in preclinical and clinical cancer studies. The chemicals consist of diethylenedihydroxyhomospermine (SBP-101), N1,N12-bis(ethyl)-cis-dehydrogenated spermine (PG-11047), and N1,N11-bis(ethyl)nordemethylspermine (BENSpm). The outcomes of BENSpm Phase I and Phase II clinical trials were unsatisfactory (99), exhibiting neurotoxicity, but PG-11047 is altered by the configuration of the central cis-double bond and demonstrates a reduced incidence of adverse clinical effects (100). PG-11047 is a second-generation unsaturated analog of the polyamine spermine, exhibiting antitumor activity against prostatic, pulmonary, colorectal, and breast malignancies (100–102). Certain clinical trials have demonstrated that the amalgamation of PG-11047 with cytotoxic and anti-angiogenic chemotherapeutic agents (gemcitabine, docetaxel, bevacizumab, erlotinib, cisplatin, 5-fluorouracil) in patients with advanced, refractory metastatic solid tumors or lymphomas sustains stable disease (103).

SBP-101 decreased cell viability in lung, pancreatic, and ovarian cancers *in vitro*, demonstrated efficacy in decelerating the growth of pancreatic tumors both *in vitro* and *in vivo*, and is presently undergoing clinical evaluation for pancreatic cancer (104). Recent advancements in nanomedicine have led to the development of nanostructures featuring polyamine analog backbones, termed nanopolyamines. Comparable nanostructures including non-polyamine backbones have demonstrated transport through endocytosis, potentially serving as a mechanism for delivering polyamine analogs that circumvents polyamine transport (105). Self-consuming prodrug nanoparticles can also be utilized for the delivery of therapeutic nucleic acids and other drug payloads (42). For instance, Nano11047, derived from PG11047, is a biodegradable prodrug nanocarrier designed to target polyamine metabolism, serving both an innovative drug formulation and a potential vehicle for nucleic acid delivery (106). Ao Yu et al. successfully synthesized bioactive polycationic prodrugs (FPaP) derived from BENSpm, partially modified with perfluoroalkyl groups. FPaP F-PaP/siRNA nanoparticles were encapsulated in hyaluronic acid (HA) to create the ternary nanoparticles HA@F-PaP/siRNA.RNA interference (RNAi) is a novel approach to cancer therapy that necessitates the use of delivery vehicles. RNA interference (RNAi) represents a novel therapeutic strategy for cancer treatment, necessitating the use of delivery vectors, whereas HA@F-PaP/siRNA improves the delivery of siRNA within tumor cells (107). Polyamine analogs are another option for cancer treatment as an alternative to polyamine synthase inhibitors. Polyamine analogs not only play a role in inhibiting polyamine synthesis, but also serve as delivery vectors to carry nucleic acids and other substances.

## 6 Strengths and limitations of polyamines-directed therapies

Nearly all cells rely on polyamines for growth and functionality. In response to heightened metabolic requirements, polyamines and their precursors are excessively present in tumors and associated microenvironments. Depletion of polyamines enhances the effectiveness of immunotherapy *in vivo*. This review provides an overview of polyamine regulation and function in tumor-associated immune cells, summarizes the processes by which polyamines regulate immune cells, and outlines pertinent therapeutic strategies. The application of polyamines in cancer treatment shows promise; nonetheless, the clinical efficacy of individual DFMOs or polyamine analogs is constrained. The clinical emphasis in polyamine research has transitioned from monotherapy to combination therapies, utilizing polyamine transporter inhibitors alongside DFMOs to effectively counteract the compensatory mechanisms of ODCs following DFMO inhibition, thereby enhancing cancer suppression. Polyamines, however, demonstrate contradictory impacts on human health, and strategies aimed at generally inhibiting them may negatively impact the activities of other organs. Moreover, several polyamines remain whose methods of action have yet to be elucidated or investigated.
